# Seven-day Green Tea Supplementation Revamps Gut Microbiome and Caecum/Skin Metabolome in Mice from Stress

**DOI:** 10.1038/s41598-019-54808-5

**Published:** 2019-12-05

**Authors:** Eun Sung Jung, Jong il Park, Hyunjoon Park, Wilhelm Holzapfel, Jae Sung Hwang, Choong Hwan Lee

**Affiliations:** 1 Department of Systems Biotechnology, Konkuk University, Seoul, 05029 Republic of Korea; 2 Department of Genetic Engineering & Graduate School of Biotechnology, Kyung Hee University, Yongin, 17104 Republic of Korea; 3 Graduate School of Advanced Green Energy and Environment, Handong Global University, Pohang, 37554 Republic of Korea; 4 Department of Bioscience and Biotechnology, Konkuk University, Seoul, 05029 Republic of Korea; 5 Research Institute for Bioactive-Metabolome Network, Konkuk University, Seoul, 05029 Korea

**Keywords:** Metabolomics, Small molecules

## Abstract

Green tea supplementation has beneficial health effects. However, its underlying mechanisms, such as effects on modulating the intestinal microbiome and endogenous metabolome, particularly following short-term supplementation, are largely unclear. We conducted an integrative metabolomics study to evaluate the effects of short-term (7-day) supplementation of green tea extract (GTE) or its components, epigallocatechin gallate, caffeine, and theanine, on the caecum microbiota and caecum/skin metabolome in mice. Further, we established an integrative metabolome-microbiome model for correlating gut and skin findings. The effects of short-term supplementation with dietary compounds were evaluated with respect to UV stress response, with GTE showing the most remarkable effects. Biplot analysis revealed that Bifidobacteria and *Lactobacillus* spp. were considerably influenced by short-term GTE supplementation, while *Clostridium butyricum* was significantly increased by UV stress without supplementation. GTE supplementation helped the skin metabolome defend against UV stress. Interestingly, a significant positive correlation was observed between caecum bacteria (Bifidobacteria, *Lactobacillus* spp.) and metabolites including skin barrier function-related skin metabolites, caecal fatty acids, and caecal amino acids. Overall, 7-day GTE supplementation was sufficient to alter the gut microbiota and endogenous caecum/skin metabolome, with positive effects on UV stress response, providing insight into the mechanism of the prebiotic effects of GTE supplementation.

## Introduction

Dietary polyphenol compounds are important substrates for the intestinal microbiota and contribute to intestinal health by modulating intestinal microbial community composition^1^. Dietary polyphenol compounds may play important roles in the growth or survival of beneficial intestinal bacteria such as *Lactobacillus* and Bifidobacteria spp., and thus exert prebiotic actions and inhibit the growth of pathogenic bacteria species^1,2^. Green tea consumption has recently been shown to influence intestinal microbiome composition. Many studies showed that green tea consumption not only alters microbial diversity and core microbiota in healthy human faecal microbiota^3^, but also increases the proportion of Bifidobacteria species in human faecal microbiota^2^. Additionally, green tea consumption has shown beneficial and disease-improving effects in previous studies of high-fat diet-induced obesity, adipocyte hypertrophy, and hepatic steatosis. These effects are highly related to the modulation of the intestinal microbiota and metabolic pathways^4,5^. Dietary polyphenol compounds also show photo-protective properties and enhance endogenous photo-protection by scavenging reactive oxygen species and modulating cellular responses or stress-dependent signaling^6^. Numerous studies have reported the photo-protective effects of green tea administration^7,8^.

In biological systems such as cells, tissues, and organs, metabolomic approaches study various small molecules. Small molecules are the final products of metabolic responses in living systems, and can be used as biomarker candidates for various disease states^9,10^. Integrated analyses of metabolomics and microbial communities have recently increased in popularity^11,12^. Merging metabolomics and microbial community analyses can provide valuable information regarding how the microbiome functions in various environments such as the gut, which may be explained by modulation of the microbial community and metabolome. Particularly, recent studies examined the interrelationship between gut and skin conditions^13,14^. Additionally, we showed that prolonged green tea supplementation influences the large intestinal microbiota and exo/endogenous metabolome in ultraviolet (UV) B-exposed mice^15^. In addition, studies on the effects of short-term green tea intake on the body have also been carried out, showing that green tea extract (GTE) can increase fat oxidation and can improve insulin sensitivity and glucose tolerance during moderate-intensity exercise in healthy young men 24 h after intake^16^. Hodgson *et al*.^17^ reported acute effects of GTE intake for 7 days in endogenous metabolites in human plasma. Based on the results of these studies, we devised the experimental scheme of this study, and thus hypothesized that short-term (7-day) supplementation of GTE modulates the intestinal microbiota and endogenous metabolome in mice, mitigating responses to UV stress. Furthermore, we also examined the effects of short-term supplementation with single compounds including epigallocatechin gallate (EGCG), caffeine, and theanine in mice compared to supplementation with GTE.

## Results

### Study design and caecum microbial community analysis

**Figure 1:**
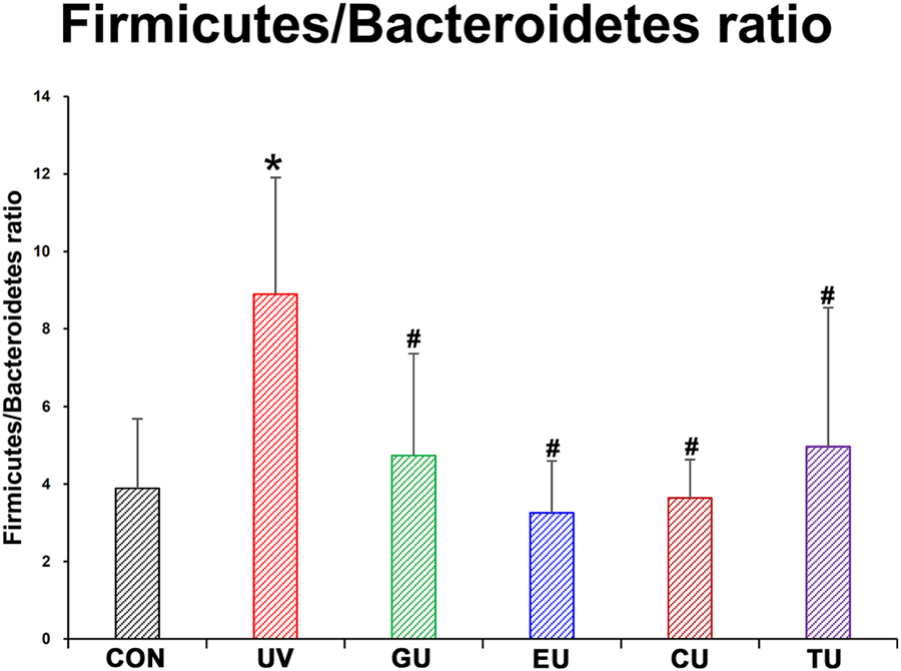
Ratio of Firmicutes to Bacteroidetes in each experimental group calculated using relative abundance of target 16S rRNA gene with a specific bacterial primer. CON (control), UV (exposure to single UV stress without supplementation), GU (7-day green tea extract supplementation followed by single UV stress), EU (7-day EGCG supplementation followed by single UV stress), CU (7-day caffeine supplementation followed by single UV stress), TU (7-day theanine supplementation followed by single UV stress). *p < 0.01 compared to CON group, ^#^*p < *0.01 compared to the UV group.

**Figure 2:**
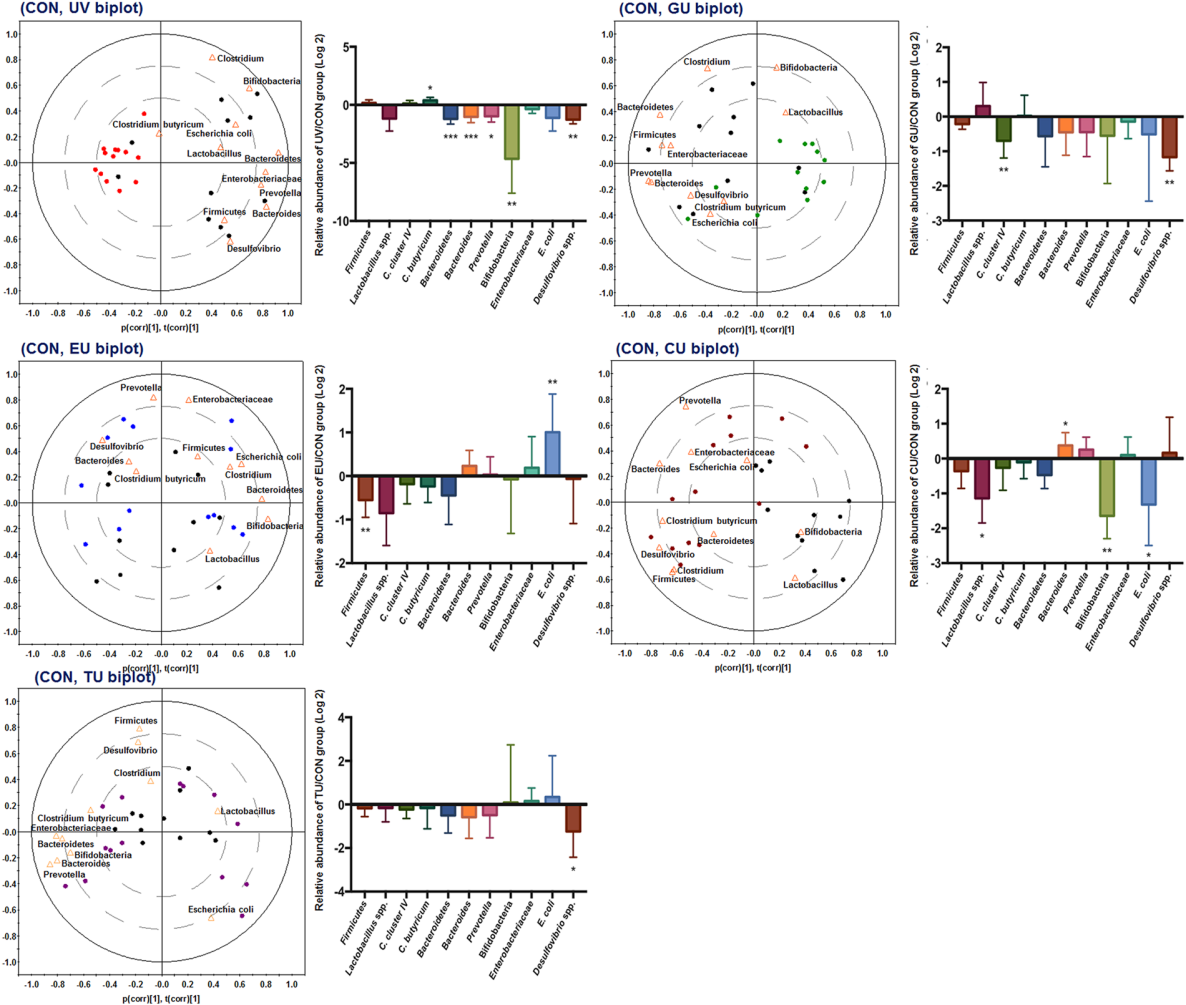
Principal component analysis (PCA) biplot analysis for caecum microbiota analysed by gut low-density array (GULDA). Each PCA biplot was generated using two groups. *Black filled circle*―CON (control), *red filled circle*―UV (exposure to single UV stress without supplementation), *green filled circle*―GU (7-day green tea extract supplementation followed by single UV stress), *blue filled circle*―EU (7-day EGCG supplementation followed by single UV stress), *brown filled circle*―CU (7-day caffeine supplementation followed by single UV stress), *purple filled circle*―TU (7-day theanine supplementation followed by single UV stress). Quantitative caecum microbial community comparison of bacterial taxa derived from GULDA. Ratio (log2) of the relative abundance of selected 16S rRNA gene targets in caecum from each group to that of the CON group.

Female Skh:HR-1 mice were subjected to comprehensive metabolomics analysis to evaluate the effects of supplementing with GTE or its components EGCG, caffeine, and theanine for 7 days prior to UV stress on the caecum microbiota and endogenous metabolome changes. Further, a correlation model was established to examine skin metabolome changes and erythema formation. To investigate the effects of short-term supplementation of GTE or its components on the caecum environment prior to UV stress in mice, the caecum microbiota of bacterial taxa derived from gut low-density array (GULDA) were quantitatively compared. The ratio of Firmicutes to Bacteroidetes, calculated as the relative abundance of the 16S rRNA gene with a specific bacterial primer, was significantly increased by UV stress without prior supplementation. In contrast, 7-day supplementation with GTE, EGCG, caffeine, or theanine significantly inhibited this increase in the ratio of Firmicutes to Bacteroidetes (Fig. 1). The fold changes in the relative abundance of bacterial taxa in the caecum from each group to the CON group are shown as bar charts (Fig. 2). Additionally, to determine the major bacterial taxa affected by supplementation with dietary compounds, we constructed principal component analysis (PCA) biplots (Fig. 2). Using bar charts and PCA biplots, we compared the effects of supplementing with dietary compounds prior to UV stress on the caecum microbial community. According to the PCA biplot of the CON and UV groups, *Clostridium butyricum* was highly correlated with the UV group, and also significantly increased in the UV group compared to the CON. Prior supplementation of dietary compounds modulated the microbial community, altering influential bacteria in each group from that in the CON group. The bacteria that differed the most in the GU group from that in the CON group were Bifidobacteria and *Lactobacillus*. The CU group showed a more diverse distribution of bacteria taxa including *Prevotella*, *Bacteroides*, *Desulfovibrio*, *Clostridium*, Firmicutes, and *C. butyricum* from the CON group. The EU and TU groups were not clearly discriminated from the CON group. These results indicate that short-term supplementation of GTE and caffeine modulate the caecum microbial community and that these changes remained even after UV stress. Short-term supplementation of EGCG and theanine also influenced the caecal microbial community, which inhibited modulations resulting from UV stress.

### Alterations in the caecum endogenous metabolome mediated by short-term dietary compound supplementation

**Figure 3:**
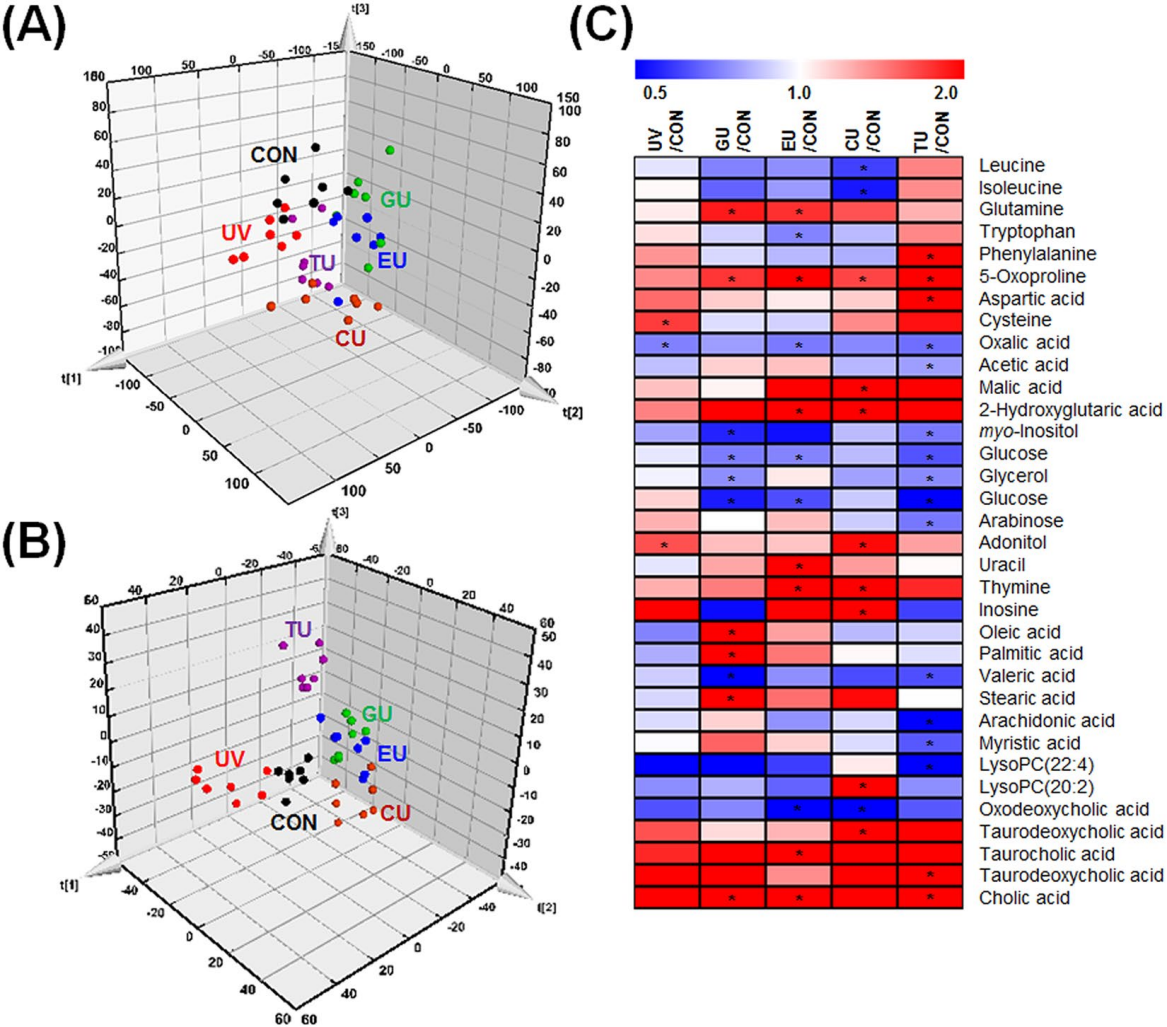
Metabolite profiling of caecum extracts based on GC-TOF-MS and UPLC-Q-TOF-MS analysis. (**A**) Three-dimensional PLS-DA score plot of GC-TOF-MS analysis. **(B**) Three-dimensional PLS-DA score plot of UPLC-Q-TOF-MS analysis. (**C**) Heatmap of significantly different metabolites (VIP > 1, *p*-value < 0.05) derived from PLS-DA (**A,B**). Each data point shown on the heatmap was normalized to the values of the CON group. *Black filled circle*―CON (control), *red filled circle*―UV (exposure to single UV stress without supplementation), *green filled circle*―GU (7-day green tea extract supplementation followed by single UV stress), *blue filled circle*―EU (7-day EGCG supplementation followed by single UV stress), *brown filled circle*―CU (7-day caffeine supplementation followed by single UV stress), *purple filled circle*―TU (7-day theanine supplementation followed by single UV stress).

To investigate the changes in the caecal metabolome associated with changes to the microbial community, endogenous caecum metabolite profiling was carried out by gas chromatography time-of-flight mass spectrometry (GC-TOF-MS) and ultraperformance-liquid chromatography quadrupole time-of-flight mass spectrometry (UPLC-Q-TOF-MS). Multivariate analysis was also performed to compare metabolite differences among each group using the partial least squares discriminant analysis (PLS-DA). According to PLS-DA score plots, the CON, UV, GU, EU, CU, and TU groups were clearly discriminated from each other (Fig. 3A,B). To select the major variables among the experimental groups, variable importance in projection (VIP) values (>1.0) of PLS-DA were used. Thirty-five caecum metabolites including amino acids, organic compounds, carbohydrates, nucleobases, lysophospholipids, and bile acids were selected and identified as significantly discriminant metabolites (Table S1). The relative levels of metabolites were normalized to CON group values and visualized using a heatmap (Fig. 3C). The heatmap showed increased levels of several amino acids, carbohydrates, nucleobases, and bile acids and decreasing patterns of fatty acids and lysophospholipids in the UV group. However, few metabolites were significantly modulated, including cysteine, oxalic acid, and adonitol, indicating that UV stress without supplementation caused mild changes in the endogenous caecum metabolome. However, short-term supplementation of GTE, EGCG, caffeine, or theanine prior to UV stress induced diverse metabolite changes in the caecum. Particularly, GTE and EGCG supplementation altered similar metabolites. The following metabolites showed remarkably higher variation in each experimental group than in the UV group: GU-glutamine, 5-oxoproline, *myo*-inositol, glucose, glycerol, oleic acid, palmitic acid, and stearic acid; EU-glutamine, tryptophan, 5-oxoproline, 2-hydroxyglutaric acid, glucose, uracil, thymine, and oxodeoxycholic acid; CU-leucine, isoleucine, 5-oxoproline, malic acid, 2-hydroxyglutaric acid, adonitol, thymine, inosine, lysoPC(20:2), and oxodeoxycholic acid; TU-phenylalanine, 5-oxoproline, aspartic acid, *myo*-inositol, glucose, glycerol, arabinose, valeric acid, arachidonic acid, and myristic acid.

### Biochemical observation of mouse dorsal skin and endogenous skin metabolome changes mediated by short-term dietary compound supplementation

**Figure 4:**
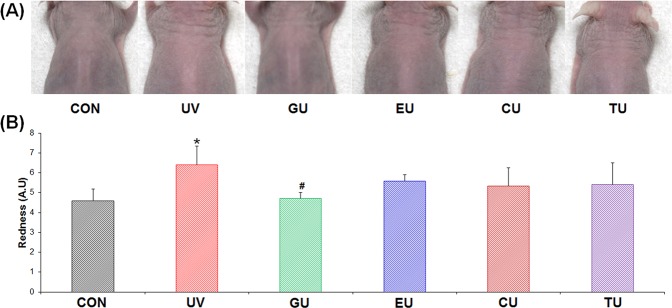
Biochemical observation of dorsal skin tissue. Erythema was measured after 24 h of UV stress. (**A**) Photograph, (**B**) skin redness (a-value). CON (control), UV (exposure to single UV stress without supplementation), GU (7-day green tea extract supplementation followed by single UV stress), EU (7-day EGCG supplementation followed by single UV stress), CU (7-day caffeine supplementation followed by single UV stress), TU (7-day theanine supplemented followed by single UV stress).*p < 0.01 compared to CON group, ^#^*p < *0.01 compared to UV group.

Erythema formation in the dorsal skin of mice was measured after 24 h of UV stress. Erythema formation in the UV groups was considerably higher than that in the CON group and slightly lower in the GU group (Fig. 4A). Differences from the UV group were unremarkable in the short-term GTE component-supplemented groups, EU, CU, and TU. Skin redness was measured with a colour reader to determine the a-value on the dorsal skin surface (Fig. 1B). Skin redness was 4.6 ± 0.58, 6.41 ± 0.94*, 4.71 ± 0.30^#^, 5.59 ± 0.30, 5.32 ± 0.93, and 5.4 ± 1.10 in the CON, UV, GU, EU, CU, and TU groups, respectively (*p < 0.01 compared to CON, ^#^*p < *0.01 compared to UV). Skin malondialdehyde (MDA) and tumour necrosis factor-α (TNF- α) levels did not significantly differ among groups (Fig. S1). These data indicate that 7-day supplementation with GTE significantly prevented UV stress-induced erythema formation, while EGCG, caffeine, and theanine showed no significant preventative effects.

**Figure 5:**
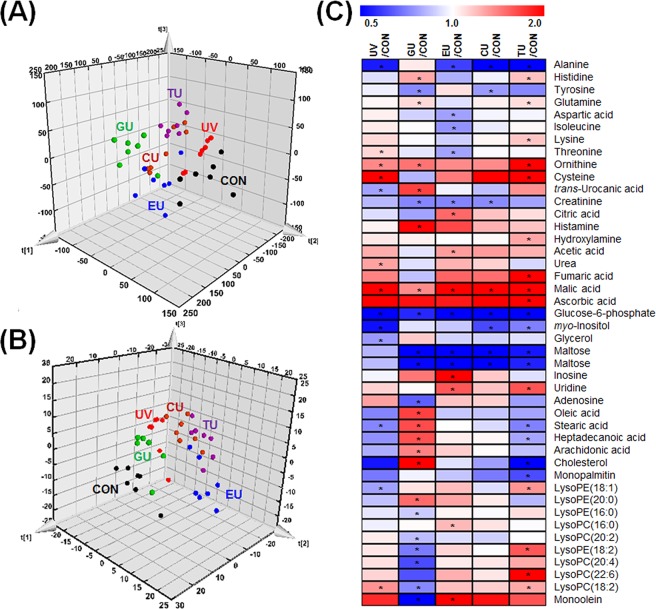
Metabolite profiling of skin tissue extracts based on GC-TOF-MS and UPLC-Q-TOF-MS analysis. (**A**) Three-dimensional PLS-DA score plot of GC-TOF-MS analysis. (**B**) Three-dimensional PLS-DA score plot of UPLC-Q-TOF-MS analysis. (**C**) Heatmap of significantly different metabolites (VIP > 1, *p*-value < 0.05) derived from PLS-DA (**A,B**). Each data point shown on the heatmap was normalized to the values of the CON group. *Black filled circle*―CON (control), *red filled circle*―UV (exposure to single UV stress without supplementation), *green filled circle*―GU (7-day green tea extract supplementation followed by single UV stress), *blue filled circle*―EU (7-day EGCG supplementation followed by single UV stress), *brown filled circle*―CU (7-day caffeine supplementation followed by single UV stress), *purple filled circle*―TU (7-day theanine supplementation followed by single UV stress).

To determine the effect of short-term supplementation with GTE or its components prior to UV stress on modulating the endogenous skin metabolome, comprehensive metabolite profiling was performed. Three-dimensional PLS-DA score plots in the CON, UV, GU, EU, CU, and TU groups were clearly discriminated from each other (Fig. 5A,B), showing similar distribution patterns to those observed from caecum metabolite profiling. Forty-four discriminant skin endogenous metabolites among experimental groups were selected based on their VIP values (>1.0) and *p* value (<0.05), and tentatively identified. Those of discriminant metabolites included 10 amino acids, 10 organic compounds, 5 carbohydrates, 3 nucleobases, 4 fatty acids, and 12 lipids. Relative metabolite levels were expressed as the fold-change ratio by normalization with the CON group and a heatmap was constructed (Fig. 5C). Further information is summarized in Supplementary Table 2. According to the heatmap, UV stress without prior dietary compound supplementation increased the levels of most amino acids, organic compounds, nucelobases, and lysophospholipids and decreased levels of carbohydrates and fatty acids (Fig. 2C). Short-term supplementation of GTE, EGCG, caffeine, or theanine resulted in different effects on the skin metabolome. In the GU group, the opposite metabolic change patterns were observed to those in the UV group including several amino acids, organic compounds, and nucleobases, as well as most fatty acids and lysophospholipids. Particularly, histidine, tyrosine, trans-urocanic acid, histamine, adenosine, oleic acid, stearic acid, heptadecanoic acid, arachidonic acid, cholesterol, lysoPE(20:0), lysoPE(16:0), lysoPC(20:2), lysoPE(18:2), lysoPC(20:4), lysoPC(18:2), and monoolein showed the most significant changes. Slight skin metabolome differences were observed in the EU, CU, and TU groups compared to the UV group. Levels of several amino acids including alanine, aspartic acid, isoleucine, and threonine were significantly decreased and levels of nucleobases (inosine and uridine) were increased in the EU group, while the CU and TU groups showed very similar metabolic distribution patterns as those of the UV groups.

### Correlations between modulation of the caecum microbial community and endogenous metabolome

**Figure 6:**
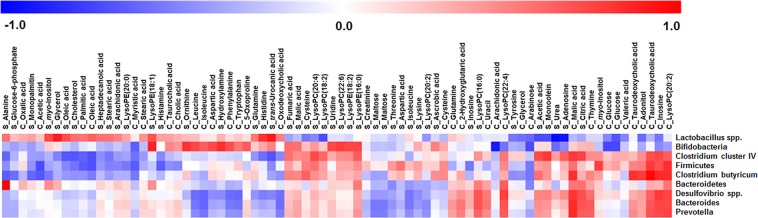
Correlation map of caecum microbiota and endogenous metabolites in skin and caecum according to Pearson’s correlation coefficient. Metabolite name starting with ‘S_’ indicates skin metabolites and those starting with ‘C_’ indicate caecum metabolites.

Correlation analysis of the caecum microbiota and endogenous metabolites in the caecum/skin was performed to determine the relationship between changes in the microbiota and metabolome in accordance with the effects of short-term dietary compound supplementation in mice (Fig. 6). Depending on the bacteria, the correlation map showed different correlation patterns in caecum and skin metabolites. Particularly, *Lactobacillus* spp. and Bifidobacteria were more distinct than the others. *Lactobacillus* spp. showed positive correlations with caecum fatty acids, amino acids, skin fatty acids, amino acids, *trans*-urocanic acid, and lipids. Furthermore, Bifidobacteria showed positive correlations with caecum amino acids and skin lysophospholipids. *Clostridium* cluster IV, Firmicutes, *C. butyricum*, *Desulfovibrio*, *Bacteroides*, and *Prevotella* showed positive correlations with various caecum metabolites including malic acid, thymine, taurodeoxycholic acid, adonitol, inosine, and lysoPC(20:2).

## Discussion

Based on previous studies, we used an animal model to examine whether supplementation with GTE or its components EGCG, caffeine, and theanine modulate the intestinal microbiota and endogenous metabolome in mice. We focused on the effects of short-term (7-day) supplementation on modulating the caecum microbiota and caecum/skin endogenous metabolome, their relationships, and skin erythema-preventing effects. Green tea has a complex chemical composition containing proteins (15–20%), carbohydrates (including cellulose and pectins, 7–25%), amino acids (including theanine, 1–4%), minerals (5%), flavonoids (including catechins, 25–35%), xanthic bases (including caffeine, 3.5%), pigments (0.5–2%), and phenolic acids (including chlorogenic acid and caffeic acid)^18,19^. Therefore, in this study, we used GTEs with very high catechin contents (50% total catechin) to minimize the effects of these minor components. In addition, single components were supplemented at higher levels to mice than those present in the green tea extracts such as 50% EGCG, 10% caffeine, and 2% theanine. When we designed the animal experiment, we expected clear effects of each component treatment. For these, we choose higher concentrations than actual concentrations in green tea. The amount of EGCG was selected based on the same amount as the total catechin content. The amount of caffeine and theanine was increased by a similar ratio to that of EGCG. However, several studies reported antioxidant activities of green tea minor components such as trace elements, phenolic acids, and pigments^18,20,21^. Thus, the effect of the green tea extract *in vivo* might be due to the synergistic effect of the major components in green tea, or might be due to the combined effect of the minor and the major components.

Highly diverse bacteria comprise the intestinal microbiota and produce various microbial metabolites with important roles in modulating the microbiome modulation. These modulations are highly affected by environmental conditions in the intestine and influence the host metabolome and health^22^. We observed that UV stress without prior supplementation notably altered the caecum microbial community, as shown by the significant increase in *C. butyricum* and decrease in Bacteriodetes, *Bacteroides*, *Prevotella*, Bifidobacteria, and *Desulfovibrio* spp. (Fig. 2). Cutaneous UV exposure disturbs the immune system of the skin through UV-induced inflammation and damages the skin, skin-associated cells, and tissues^23^. Additionally, UV exposure affects bacterial cells. A previous study showed that the viability of *Lactobacillus* spp. decreased immediately upon UV exposure, but was restored over time and enhanced cholesterol removal ability^24^. We previously showed that prolonged UVB exposure induced marked changes in the microbiota of the large intestine^15^. Thus, UV stress appears to influence the intestinal bacteria. In our study, *C. butyricum* was significantly increased in the UV group. Few studies have examined the relationship between UV stress and *C. butyricum* levels. *C. butyricum* is a gram-positive, spore-forming, butyric acid-producing, and obligate anaerobic species found in faeces^25^. Indigenous spore-forming bacteria have various cell morphologies in the intestine such as resting spores, germinated spores, vegetative cells, and endotrophic cells^25^. Bacterial spores are even more resistant to chemicals, heat, and irradiation than their respective vegetative cells^26^. In addition, studies have shown that *C. butyricum* is resistant to various antibiotics and UV rays^27^. Particularly, *Clostridium* spp. show higher d-values, representing the decimal reduction time required at a given condition to kill 90% of the exposed microorganisms, than gram-negative bacteria^28^. Among the bacterial groups we analysed, Bacteroidetes, *Bacteroides*, *Prevotella*, Enterobacteriaceae, *E. coli*, and *Desulfovibrio* spp. are gram-negative. Among gram-positive bacteria, *Clostridium* spp. are the only spore-forming bacteria. Thus, *C. butyricum* might be relatively more resistant to single UVB irradiation than other bacteria, which leads to relatively high bacterial counts. Some studies also showed that *C. butyricum* has various uses as an antibacterial agent, in gut epithelial cell proliferation, and in ulcerative colitis therapy^29–31^. Unlike in the UV group, short-term supplementation with GTE showed different caecum microbiota modulation patterns including significantly decreased *Clostridium* cluster IV and *Desulfovibrio* spp. According to the PCA biplot, the most influential bacteria distinguishing the GU group from the CON group were *Lactobacillus* spp. and Bifidobacteria (Fig. 2). In agreement with previous studies, we observed increased levels of *Lactobacillus* spp. and *Bifidobacterium* spp. in human faecal samples upon green tea consumption^1,2^. Additionally, oral administration of probiotics such as *Lactobacillus* and *Bifidobacterium* species was previously shown to have photo-protective effects on UV-exposed skin^32–35^. Furthermore, profound correlations were observed between other skin diseases such as atopic dermatitis and intestinal probiotics^36,37^. Particularly, colonization of probiotics in the intestine was associated with a decrease in the disease state^36,38^. The PCA biplot of the CU and CON groups also showed clear discriminant patterns; levels of *Lactobacillus* spp. and Bifidobacteria were considerably lower in the CU group than levels in the CON group. Overall, these results and results of previous studies suggest that 7-day supplementation with GTE modulates sustained changes in the caecum microbial community, particularly *Lactobacillus* spp. and Bifidobacteria, and that these changes might prevented UV stress-induced modulation of the microbial community.

In metabolomics studies, sample preparation is extremely important because the metabolome composition could be affected by numerous factors, such as time, method of sampling, environment, and how the sample is stored. Before metabolite extraction, accurate sampling and quenching procedures are required to provide a snapshot of the metabolome and determine the repeatability of the experiment^39^. To provide exact metabolomics data, samples must be accurately prepared. We observed diverse skin metabolome changes following exposure to UV stress. In the GU group, the opposite metabolic change patterns were observed from those in the UV group, particularly for histidine, tyrosine, *trans*-urocanic acid, histamine, adenosine, oleic acid, stearic acid, heptadecanoic acid, arachidonic acid, cholesterol, lysoPE(20:0), lysoPE(16:0), lysoPC(20:2), lysoPE(18:2), lysoPC(20:4), lysoPC(18:2), and monoolein (Fig. 5C). These amino acids, urocanic acid, fatty acids, and lipids are closely related to skin barrier functions. Urocanic acid, which is found in the stratum corneum of the epidermis, plays an important role in protecting the epidermis from UVB-induced damage^40^. Free fatty acids are very actively synthesized in the epidermis, but are decreased by acute UV irradiation accompanied by decreased expression of lipid synthesis-related genes^41,42^. Cholesterol synthesis is also highly related to the recovery of the permeability barrier function of the skin^42^. Lysophospholipids are among the most important factors in skin barrier function, skin hydration, and skin inflammation^43,44^. We also observed significant preventive effects on erythema formation in only the GTE-supplemented group (Fig. 4). These differences in preventing erythema formation may be related to the intake level or characteristics of the supplements. According to other reports, both oral administration and topical application of green tea can reduce UVB irradiation-induced erythema in skin *i.e*., consuming green tea polyphenols of human (1402 mg/day, 6 weeks), oral administration of green tea extracts in mice (250 mg/kg. day, 12 weeks), treatment of green tea extracts on human skin (10 mg, 30 min before UV irradiation), and topical application of dermal gels containing green tea extracts (3 g/100 g gel)^45–48^. However, it remains controversial whether erythema formation is inhibited by EGCG administration. Jeon *et al*.^49^ reported that oral EGCG supplementation for 8 weeks significantly increased minimal erythema dose and attenuated UVB-induced skin damage, whereas Chow *et al*.^50^ found that administering a high daily bolus dose of EGCG for 4 weeks did not protect against UV-induced erythema. These differences may be related to the different conditions of the experiments such as supplementation amount and period. Reports substantiating the relationship between caffeine or theanine administration and anti-erythema forming effects are lacking. Instead, caffeine was shown to inhibit UV-induced carcinogenesis^8,51^, while theanine showed various bioactivities such as neuroprotective, antioxidant, anti-inflammatory effects, and improved immunity^52^. Thus, our results showed that 7-day prior supplementation of GTE, rather than single compounds (EGCG, caffeine, or theanine), significantly modulated various skin metabolites related to skin barrier function. This modulation inhibited skin metabolome changes according to UV stress, and the effects may help prevent skin erythema formation. On the other hand, no significant differences in MDA and TNF-α levels were observed among groups. MDA is a product of lipid peroxidation which is used as an oxidative stress indicator and TNF-α facilitates the migration of inflammatory cells. According to other animal studies that used similar experimental conditions as those used in our study, similar phenomena were observed in MDA and TNF-α measurements. Svobodova *et al*.^53^ measured MDA levels in hairless mice skin homogenates after exposure to a single dose of UVB (200 or 800 mJ/cm^2^) at a 45-cm distance. At both 4 h and 24 h after UVB (200 mJ/cm^2^) exposure, no significant differences in MDA levels were found compared to non-irradiated controls, but significantly decreased 24 h afterward with a higher UVB dose (800 mJ/cm^2^). Sharma *et al*.^54^ measured TNF-α mRNA levels in mice irradiated with UVB (100 mJ/cm^2^ day) for 5 days. At 3 h after the last UVB irradiation, levels of TNF-α mRNA significantly decreased, whereas no difference from non-irradiated controls was observed after 20 h. However, the MDA levels of mice skin that were exposed to a single dose of UVB (240 mJ/cm^2^) at a 7-cm distance showed significant increase^53^. Overall, results of our study and previous studies showed that the levels of MDA and TNF-α in mouse skin were induced by UVB irradiation, but results were highly influenced by irradiation conditions such as dose, distance from device, and the amount of time passed after exposure.

The relationship between the gut and skin was previously not well-understood. Microbial compounds including cell wall fragments, dead cells, and microbial metabolites may induce immune responses that improve skin health^55^. Endogenous caecum metabolite profiling was performed to evaluate the relationship between the caecum microbiota and endogenous skin metabolites. According to the heatmap of the caecum metabolome, a few metabolites were significantly altered by UV stress. However, short-term supplementation with GTE, EGCG, caffeine, or theanine induced more diverse metabolite modulations in the caecum (Fig. 3C). This may be because diet is a crucial factor affecting the composition of the intestinal microbiota, and thus each compound influenced the host in a different manner^56^. According to the heat map, bile acid levels were much higher in all treatment groups than in the CON group. Bile acids are well-known gut bacteria metabolites with various biological functions including absorbing lipid-soluble vitamins; facilitating lipid absorption; maintaining intestinal barrier function; and regulating triglyceride, cholesterol, glucose, and energy homeostasis^57^. Bacteria including *Bacteroides*, *Clostridium*, *Lactobacillus*, *Bifidobacterium*, *Enterobacter*, and *Eubacterium* are associated with bile acid metabolism^22^. These highly increased bile acids levels may be related to UV stress. In the GU group, levels of several metabolites such as glutamine, 5-oxoproline, *myo*-inositol, glucose, glycerol, oleic acid, palmitic acid, and stearic acid were altered. These metabolites showed a high correlation with the caecum probiotic bacteria *Lactobacillus* spp. and Bifidobacteria. Thus, modulation of the *Lactobacillus* spp. and Bifidobacteria populations and the effects of GTE supplementation on mice intestine appear to be closely related. These relationships may affect erythema formation in the skin.

In conclusion, we conducted comprehensive metabolome analysis to elucidate the effects of short-term (7-day) supplementation of dietary components on modulating the gut and endogenous metabolomes. Among the supplements examined, including GTE, EGCG, caffeine, and theanine, only short-term supplementation with GTE remarkably modulated the microbiome-metabolome accompanied with significant prevention of UV stress in mice. Particularly, probiotics including Bifidobacteria and *Lactobacillus* spp. were highly associated with GTE supplementation and bacteria levels were highly correlated with skin barrier function-related metabolites. Overall, 7-day supplementation of GTE effectively modulated the caecum microbiota and endogenous caecum/skin metabolome in mice; these modulations may act as indirect prebiotics and showed positive effects on preventing external UV stress.

## Methods

### Chemicals

Water, methanol, and acetonitrile were purchased from Fisher Scientific (Waltham, MA, USA). Pyridine, formic acid, *N*-methyl-*N*-(trimethylsilyl)-trifluoroacetamide, methoxyamine hydrochloride, EGCG, caffeine, and theanine were obtained from Sigma Chemical Co. (St. Louis, MO, USA). The analytical grade of chemicals were used. Green tea extract (GTE) was obtained from AmorePacific Corp. (Yongin, Korea) and contains catechins (49.9%), including epigallocatechin (EGC, 9.7%), epicatechin (EC, 5.4%), epigallocatechin-gallate (EGCG, 28.4%), and epicatechin-gallate (ECG, 6.4%), as well as caffeine (4.5%) and theanine (0.4%).

### Animal experiments

Six-week-old female Skh-1 mice were purchased from Orient Bio (Seongnam, Korea). Mice were housed under controlled conditions as follows: diet (Ain-93G), temperature (24 ± 2 °C), light (12-h light/dark cycle), and humidity (55 ± 10%). The mice were randomly divided into six experimental groups (n = 7, each group) after one week of acclimation: Control (CON), exposure to single UV stress without supplementation group (UV), 7-day GTE supplementation followed by single UV stress group (GU; 6.7 mg/day), 7-day EGCG supplementation followed by single UV stress group (EU; 3.35 mg/day), 7-day caffeine supplementation followed by single UV stress group (CU; 0.67 mg/day), and 7-day theanine supplementation followed by single UV stress group (TU; 0.134 mg/day). For the single exposure to UV stress, four fluorescent lamps (TL 20 W/12 RS SLV) were used with peak emission of 315 nm and wavelength of 290–390 nm (Philips, Amsterdam, Netherlands). Intensity was monitored using a UV meter (v.2.03, VARIOCONTROL; Waldmann, Villingen-Schwenningen, Germany). Mice were exposed to UVB irradiation after 7 days of supplementation with each compound by moving freely in the cage at 30 cm from the lamps (188 mJ/cm^2^). After 24 h of UV stress, the mice were sacrificed, and their dorsal skin and caecum tissues were taken and stored in a deep freezer (−80 °C). The protocols of animal experiments were approved by the Institutional Animal Care and Use Committee of Gyeonggi Institute of Science & Technology. The experimental methods were also performed in accordance with their guidelines and regulations (permit number: 2016–03–0007).

### Microbial analysis of caecum

The caecum microbiota was analysed by real-time PCR-based gut low-density array (GULDA)^58^. Cellular DNA extraction was conducted using the QIAGEN Stool mini kit (Hilden, Germany) according to the manufacturer’s instructions (except inhibitor EX), including bead-beating steps (0.1, 1 mm zirconium). Relative quantification of 16 s rRNA gene target primers was conducted in the following bacterial groups: Firmicutes, *Lactobacillus* spp., *Clostridium* cluster IV, *Clostridium butyricum*, Bacteroidetes, *Bacteroides*, *Prevotella*, Bifidobacteria, Enterobacteriaceae, *Escherichia coli*, and *Desulfovibrio* spp.

### Sample preparation and MS-based metabolite profiling

For metabolite extraction, finely chopped skin tissues (2 × 3 cm) and caecum samples stored in the deep freezer were used immediately. Samples were homogenized in 1 mL of methanol using an MM400 mixer mill (Retch, Haan, Germany) with a frequency of 30 s^−1^ for 10 min using zirconium beads. The homogenates were kept at 4 °C for 60 min, then centrifuged (12,578 × *g*, 4 °C, 10 min). The filtered (0.2-μm polytetrafluoroethylene filter) supernatants were dried under vacuum using a speed-vacuum concentrator (Biotron, Gyeonggi-Do, Korea). The chamber temperature of speed-vacuum concentrator was set at 30 °C. Dried extracts were further analysed by two different MS-based methods i.e., GC-TOF MS and UPLC-Q-TOF-MS. During MS analysis, samples were analysed randomly. The operational conditions for GC-TOF-MS analysis and UPLC-Q-TOF-MS were described in our previous study^59^.

### Clinical observation of skin tissues

The dorsal skin of hairless mice was photographed before sacrifice. The erythema index of the dorsal skin of hairless mice was measured using a Mexameter 18 and Corneometer 825 before sacrifice.

### Data processing and multivariate statistical analysis

The data processing procedure of MS-derived raw data, multivariate analysis method, and metabolite identifications were performed as described in our previous study^60^. A correlation map was constructed using the MeV software package (http://www.tm4.org/) based on correlation coefficient values calculated with PASW Statistics 18 software.

## Supplementary information

raw data

Supplementary Information

## Data Availability

All data generated or analysed during this study are included in this published article and its Supplementary Information Files.
