# Supplementary material for: Seven-day Green Tea Supplementation Revamps Gut Microbiome and Caecum/Skin Metabolome in Mice from Stress
Source: Sci Rep. 2019 Dec 5;9:18418. doi: 10.1038/s41598-019-54808-5 (PMC6895175; doi:10.1038/s41598-019-54808-5)

**Seven-day Green Tea Supplementation Revamps Gut Microbiome and Caecum/Skin Metabolome in Mice from Stress**

Eun Sung Jung^1^, Jong il Park^2^, Hyunjoon Park^3^, Wilhelm Holzapfel^3^, Jae Sung Hwang^2,*^, and Choong Hwan Lee^1,4,5*^

^1^ Department of Systems Biotechnology, Konkuk University, Seoul 05029, Republic of Korea

^2^ Department of Genetic Engineering & Graduate School of Biotechnology, Kyung Hee University, Yongin 17104, Republic of Korea

^3^ Graduate School of Advanced Green Energy and Environment, Handong Global University, Pohang 37554, Republic of Korea

^4^ Department of Bioscience and Biotechnology, Konkuk University, Seoul 05029, Republic of Korea

^5^ Research Institute for Bioactive-Metabolome Network, Konkuk University, Seoul 05029, Korea

*** Co-corresponding Authors**

**Jae Sung Hwang, Ph. D.**

Department of Genetic Engineering and Graduate School of Biotechnology, Kyung Hee University, 1732 Deogyeong-daero, Giheung-gu, Yongin 17104, Republic of Korea
Tel.: +82-31-888-6179, Fax: +82-31-888-6173, E-mail: jshwang@khu.ac.kr

**Choong Hwan Lee, Ph. D.**

Department of Systems Biotechnology, Konkuk University, 120 Neungdong-ro, Gwangjin-gu, Seoul 05029, Republic of Korea

Tel.: +82-2-2049-6177, Fax: +82-2-455-4291, E-mail: chlee123@konkuk.ac.kr

**Supplementary Table 1.** Discriminate endogenous metabolites in caecum modulated by UV stress or short-term administration of each supplements analyzed by GC-TOF-MS and UPLC-Q-TOF-MS.

| GC-TOF-MS | | | | | UPLC-Q-TOF-MS | | | | | | |
| --- | --- | --- | --- | --- | --- | --- | --- | --- | --- | --- | --- |
| Metabolites^a^ | RT^b^  (min) | Identified  ion (*m/z*) | MS fragment | ID^c^ | Metabolites^a^ | RT^b^  (min) | Identified  ion (*m/z*) | Adduct | Molecular  formular | PPM^d^ | ID^c^ |
| *Amino acids* | | | | | *Bile acids* | | | | | | |
| Leucine | 7.21 | 158 | 59,73,74,75,100,102,147,158,159,160 | STD/MS | Taurocholic acid^e^ | 5.38 | 514.2832 | [M-H]^-^ | C26H45NO7S | -1.6 | Lib. |
| Isoleucine | 7.43 | 158 | 57,73,74,75,136,147,158,159,180,218 | STD/MS | Taurodeoxycholic acid^f^ | 5.51 | 498.2882 | [M-H]^-^ | C26H45NO6S | -2.8 | Lib. |
| Aspartic acid | 9.45 | 232 | 61,73,74,75,100,128,147,176,232,233 | STD/MS | Cholic acid^g^ | 5.80 | 407.2790 | [M-H]^-^ | C24H40O5 | -2.5 | Lib. |
| 5-Oxoproline | 9.50 | 156 | 56,73,74,75,84,147,156,157,158,174 | STD/MS | Taurodeoxycholic acid^f^ | 6.17 | 498.2887 | [M-H]^-^ | C26H45NO6S | -1.6 | Lib. |
| Cysteine | 9.75 | 220 | 59,73,74,75,84,100,132,147,218,220 | STD/MS | Oxodeoxycholic acid | 6.43 | 405.2635 | [M-H]^-^ | C24H38O5 | -0.2 | Lib. |
| Phenylalanine | 10.34 | 218 | 73,74,75,100,147,192,193,218,219,220 | STD/MS | *Lipids* |  |  |  |  |  |  |
| Glutamine | 11.41 | 226 | 73,74,75,100,147,156,157,218,226 | STD/MS | LysoPC(22:4) | 8.78 | 556.3400 | [M-CH_3_]^-^ | C30H54NO7P | 2.0 | Lib. |
| Tryptophan | 14.37 | 202 | 73,74,75,131,147,202,203,204,218,291 | STD/MS | LysoPC(20:2) | 9.11 | 532.3411 | [M-CH_3_]^-^ | C28H54NO7P | -1.1 | Lib. |
| *Organic compounds* | | | | |  |  |  |  |  |  |  |
| Acetic acid | 5.16 | 177 | 59,66,73,74,75,133,147,148,149,177 | STD/MS |  |  |  |  |  |  |  |
| Oxalic acid | 5.88 | 133 | 59,72,73,74,75,86,100,133,147,148 | STD/MS |  |  |  |  |  |  |  |
| Malic acid | 9.18 | 233 | 55,59,73,74,75,101,133,147,148,233 | STD/MS |  |  |  |  |  |  |  |
| 2-Hydroxyglutaric acid | 9.87 | 129 | 73,74,75,85,129,133,147,157,203,247 | MS |  |  |  |  |  |  |  |
| *Carbohydrates* | | | | |  |  |  |  |  |  |  |
| Glycerol | 7.23 | 205 | 73,74,75,103,117,147,148,149,205,218 | STD/MS |  |  |  |  |  |  |  |
| myo-Inositol | 13.63 | 191 | 73,74,75,133,147,191,217,218,305,318 | STD/MS |  |  |  |  |  |  |  |
| Arabinose | 10.64 | 103 | 73,74,75,103,104,133,147,217,218,307 | STD/MS |  |  |  |  |  |  |  |
| Adonitol | 11.07 | 217 | 73,74,75,103,117,129,147,157,160,217 | STD/MS |  |  |  |  |  |  |  |
| Glucose | 12.38 | 205 | 73,74,103,129,147,157,160,205,217,319 | STD/MS |  |  |  |  |  |  |  |
| Glucose | 12.51 | 205 | 73,74,75,103,129,147,157,160,205,319 | STD/MS |  |  |  |  |  |  |  |
| *Nucelobases* | | | | |  |  |  |  |  |  |  |
| Uracil | 7.86 | 241 | 73,99,100,113,126,147,241,242,255,256 | STD/MS |  |  |  |  |  |  |  |
| Thymine | 8.44 | 255 | 59,73,100,113,120,140,147,255,256,270 | MS |  |  |  |  |  |  |  |
| Inosine | 16.28 | 217 | 73,74,75,103,147,193,217,230,245,281 | STD/MS |  |  |  |  |  |  |  |
| *Fatty acids* | | | | |  |  |  |  |  |  |  |
| Valeric acid | 4.07 | 159 | 55,61,73,74,75,76,77,117,132,159 | MS |  |  |  |  |  |  |  |
| Myristic acid | 11.84 | 285 | 55,73,74,75,117,129,131,132,145,285 | STD/MS |  |  |  |  |  |  |  |
| Palmitic acid | 13.14 | 313 | 55,73,74,75,117,118,129,131,132,145 | STD/MS |  |  |  |  |  |  |  |
| Oleic acid | 14.20 | 339 | 55,67,73,75,81,84,96,117,129,145 | STD/MS |  |  |  |  |  |  |  |
| Stearic acid | 14.33 | 341 | 55,73,74,75,117,118,129,131,132,145 | STD/MS |  |  |  |  |  |  |  |
| Arachidonic acid | 15.09 | 91 | 55,67,73,75,77,79,80,91,93,117 | STD/MS |  |  |  |  |  |  |  |
| ^a^ Metabolites selected by VIP > 0.7 from PLS-DA models | | | | | | | | | | | |
| ^b^ Retention time | | | | | | | | | | | |
| ^c^ Identification of metabolites, STD; commercial standard compounds, MS and Lib.; in-house library | | | | | | | | | | | |
| ^d^ Mass tolerance from elemental composition analysis of MassLynx software | | | | | | | | | | | |
| ^e^ Identified as taurine conjugated with cholic acid affiliation (taurocholic acid or tauro-muricholic acid or tauroallocholic acid or taurohyocholic acid or tauroursocholic acid) | | | | | | | | | | | |
| ^f^ Identified as taurine conjugated with deoxycholic acid affiliation (taurodeoxycholic acid or tauroursodeoxycholic acid or taurochenodeoxycholic acid) | | | | | | | | | | | |
| ^g^ Identified as cholic acid affiliation (cholic acid or muricholic acid or lithocholic acid) | | | | | | | | | | | |

**Supplementary Table 2.** Discriminate endogenous metabolites in skin modulated by UV stress or short-term administration of each supplements analyzed by GC-TOF-MS and UPLC-Q-TOF-MS.

| GC-TOF-MS | | | | | UPLC-Q-TOF-MS | | | | | | |
| --- | --- | --- | --- | --- | --- | --- | --- | --- | --- | --- | --- |
| Metabolites^a^ | RT^b^  (min) | Identified  ion (*m/z*) | MS fragment | ID^c^ | Metabolites^a^ | RT^b^  (min) | Identified  ion (*m/z*) | Adduct | Molecular  formular | PPM^d^ | ID^c^ |
| *Amino acids* | | | | | *Lipids* |  |  |  |  |  |  |
| Alanine | 8.65 | 248 | 59,73,86,100,130,133,147,174,248,249 | STD/MS | LysoPE(18:1) | 8.85 | 478.2929 | [M-H]^-^ | C23H46NO7P | 0.6 | Lib. |
| Histidine | 12.49 | 154 | 73,74,75,100,103,147,154,155,218,254 | STD/MS | LysoPE(20:0) | 9.16 | 494.3252 | [M-CH_3_]^-^ | C25H52NO7P | -1.2 | Lib. |
| Tyrosine | 12.22 | 179 | 55,73,74,75,103,147,157,173,179,180 | STD/MS | LysoPE(16:0) | 8.60 | 452.2770 | [M-H]^-^ | C21H44NO7P | 2.7 | Lib. |
| Glutamine | 11.43 | 156 | 73,74,75,100,131,147,155,156,157,245 | STD/MS | LysoPC(16:0) | 8.62 | 480.3088 | [M-CH_3_]^-^ | C24H50NO7P | -0.2 | Lib. |
| Aspartic acid | 9.46 | 232 | 61,73,74,75,100,128,147,156,232 | STD/MS | LysoPC(20:2) | 9.10 | 532.3376 | [M-CH_3_]^-^ | C28H54NO7P | 2.6 | Lib. |
| Isoleucine | 7.44 | 158 | 59,73,74,75,100,142,147,158,159,218 | STD/MS | LysoPE(18:2) | 8.17 | 476.2774 | [M-H]^-^ | C23H44NO7P | 0.4 | Lib. |
| Lysine | 12.45 | 174 | 59,73,74,86,100,128,147,156,174,230 | STD/MS | LysoPC(20:4) | 8.18 | 528.3091 | [M-CH_3_]^-^ | C28H50NO7P | 2.3 | Lib. |
| Threonine | 8.32 | 219 | 57,73,75,100,101,117,129,147,218,219 | STD/MS | LysoPC(22:6) | 8.12 | 552.3099 | [M-CH_3_]^-^ | C30H50NO7P | 1.3 | Lib. |
| Ornithine | 11.74 | 142 | 59,73,74,75,86,100,142,143,147,174 | STD/MS | LysoPC(18:2) | 8.20 | 504.3090 | [M-CH_3_]^-^ | C26H50NO7P | -0.8 | Lib. |
| Cysteine | 9.76 | 220 | 59,73,75,84,100,113,115,143,147,220 | STD/MS |  |  |  |  |  |  |  |
| *Organic compounds* | | | | |  |  |  |  |  |  |  |
| *trans*-Urocanic acid | 12.97 | 267 | 73,74,75,81,147,165,180,193,267,282 | STD/MS |  |  |  |  |  |  |  |
| Creatinine | 9.80 | 115 | 59,73,74,100,115,116,143,147,171,329 | STD/MS |  |  |  |  |  |  |  |
| Citric acid | 11.78 | 273 | 67,73,74,75,133,147,148,149,157,273 | STD/MS |  |  |  |  |  |  |  |
| Histamine | 12.14 | 174 | 59,73,74,86,100,130,174,175,226,312 | STD/MS |  |  |  |  |  |  |  |
| Hydroxylamine | 5.62 | 133 | 59,72,73,86,102,119,130,133,146,147 | STD/MS |  |  |  |  |  |  |  |
| Acetic acid | 5.18 | 177 | 59,66,73,74,75,79,133,147,148,177 | STD/MS |  |  |  |  |  |  |  |
| Urea | 6.95 | 189 | 59,66,73,74,75,87,147,148,171,189 | STD/MS |  |  |  |  |  |  |  |
| Fumaric acid | 7.81 | 245 | 73,75,99,126,133,147,148,241,245,255 | STD/MS |  |  |  |  |  |  |  |
| Malic acid | 9.10 | 233 | 55,59,73,74,75,101,133,147,148,233 | STD/MS |  |  |  |  |  |  |  |
| Ascorbic acid | 12.70 | 117 | 73,74,75,103,117,133,147,148,205,217 | STD/MS |  |  |  |  |  |  |  |
| *Carbohydrates* | | | | |  |  |  |  |  |  |  |
| Glucose-6-phosphate | 15.04 | 387 | 73,74,75,129,133,147,160,217,299,387 | STD/MS |  |  |  |  |  |  |  |
| *myo*-Inositol | 13.65 | 191 | 73,74,103,129,133,147,191,217,305,318 | STD/MS |  |  |  |  |  |  |  |
| Glycerol | 7.24 | 205 | 59,73,74,75,103,117,133,147,205,299 | STD/MS |  |  |  |  |  |  |  |
| Maltose | 17.28 | 361 | 73,103,117,129,147,169,204,205,217,361 | STD/MS |  |  |  |  |  |  |  |
| Maltose | 17.42 | 361 | 73,75,103,117,129,147,169,204,217,361 | STD/MS |  |  |  |  |  |  |  |
| *Nucelobases* | | | | |  |  |  |  |  |  |  |
| Inosine | 16.31 | 281 | 73,74,75,103,147,193,217,230,245,281 | STD/MS |  |  |  |  |  |  |  |
| Uridine | 15.65 | 169 | 73,74,75,103,147,169,217,224,243,245 | STD/MS |  |  |  |  |  |  |  |
| Adenosine | 16.62 | 236 | 58,71,73,74,75,103,129,147,217,230 | STD/MS |  |  |  |  |  |  |  |
| *Fatty acids* | | | | |  |  |  |  |  |  |  |
| Oleic acid | 14.22 | 339 | 55,67,73,75,81,84,96,117,129,145 | STD/MS |  |  |  |  |  |  |  |
| Stearic acid | 14.35 | 341 | 55,57,73,75,116,117,129,131,132,145 | STD/MS |  |  |  |  |  |  |  |
| Heptadecanoic acid | 13.76 | 117 | 55,73,75,85,116,117,129,131,132,145 | STD/MS |  |  |  |  |  |  |  |
| Arachidonic acid | 15.11 | 91 | 55,59,67,72,73,75,79,80,91,117 | STD/MS |  |  |  |  |  |  |  |
| *Lipids* | | | | |  |  |  |  |  |  |  |
| Cholesterol | 19.84 | 129 | 55,57,73,75,81,91,95,105,707,129 | STD/MS |  |  |  |  |  |  |  |
| Monopalmitin | 16.25 | 371 | 55,57,73,75,103,116,117,129,147,203 | STD/MS |  |  |  |  |  |  |  |
| Monoolein | 17.09 | 129 | 55,67,73,75,81,103,129,147,204 | STD/MS |  |  |  |  |  |  |  |
| ^a^ Metabolites selected by VIP > 0.7 from PLS-DA models | | | | | | | | | | | |
| ^b^ Retention time | | | | | | | | | | | |
| ^c^ Identification of metabolites, STD; commercial standard compounds, MS and Lib.; in-house library | | | | | | | | | | | |
| ^d^ Mass tolerance from elemental composition analysis of MassLynx software | | | | | | | | | | | |

**Supplementary Figure 1.** Biochemical parameters of dorsal skin tissues of each experimental group. (A) Malondialdehyde, (B) tumor necrosis factor-alpha.


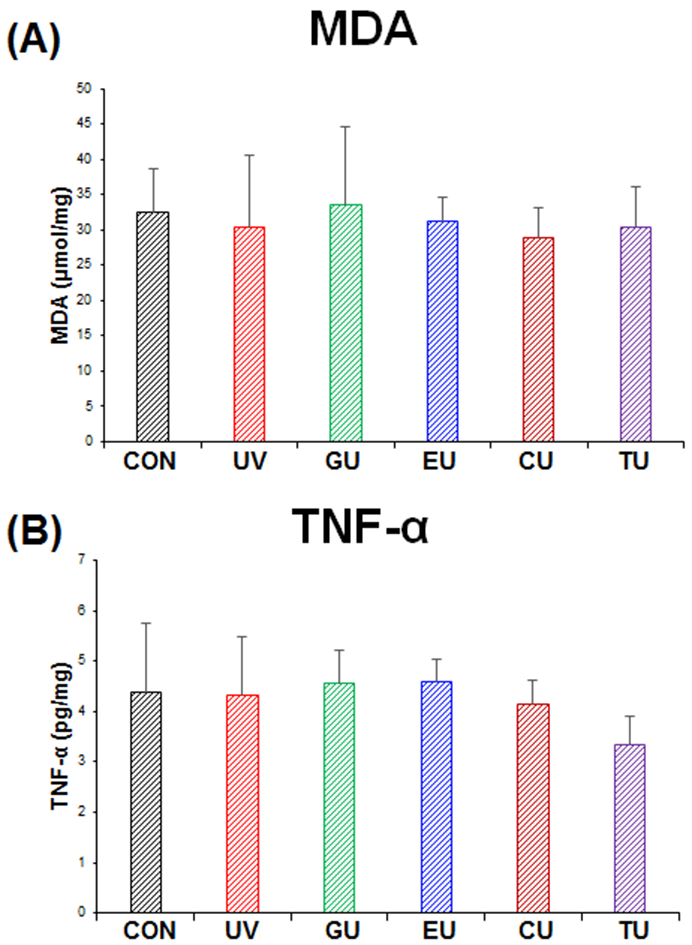

Supplement: Supplementary file 2 — Supplementary Information [file 41598_2019_54808_MOESM2_ESM.docx]
